# Three-Dimensional Evaluation of Condyle-Glenoid Fossa Complex Following Treatment with Herbst Appliance

**DOI:** 10.3390/jcm10204730

**Published:** 2021-10-15

**Authors:** Jasmine Nindra, Maninder Singh Sidhu, Anuraj Singh Kochhar, Ashish Dabas, Rosa Valletta, Roberto Rongo, Gianrico Spagnuolo

**Affiliations:** 1 Department of Orthodontics, Faculty of Dental Sciences, SGT University, Gurgaon 122505, India; jasmine_dental@sgtuniversity.org (J.N.); drmssidhu@hotmail.com (M.S.S.); ashishdabas79@gmail.com (A.D.); 2 Faculty of Dentistry, University of Toronto, Toronto, ON M5G 0C1, Canada; anuraj_kochhar@yahoo.co.in; 3 Department of Neurosciences, Reproductive and Odontostomatological Sciences, Federico II University of Naples, 80131 Naples, Italy; valletta@unina.it (R.V.); roberto.rongo@unina.it (R.R.); 4 Department of Therapeuthic Dentistry, I.M. Sechenov First Moscow State Medical University, 119991 Moscow, Russia

**Keywords:** skeletal Class II malocclusion, Herbst, CBCT, condylar volume, condyle-glenoid fossa complex

## Abstract

The purpose of the present retrospective observational study was to compare the effects of treatment with Herbst appliance and fixed therapy with elastics on the condyle and glenoid fossa complex. Thirty patients aged between twelve and sixteen years with skeletal Class II malocclusion who met the inclusion criteria were included in the study: fifteen patients treated with Herbst appliance (Group 1), and fifteen patients treated with orthodontic camouflage using MBT prescription (MBT^TM^ Versatile+ Appliance System) (Group 2). For Group 2, patients had CBCT scans taken before treatment either after Herbst appliance removal or at the end of treatment. CBCT scans were evaluated for changes in condyle-glenoid fossa complex using the In Vivo Dental 5.1 software. Statistical significance was set at *p* ≤ 0.05. On inter-group comparison, the Herbst group showed statistically significant increases in the condylar height of 1.35 mm (*p* ≤ 0.001) on the right and 1.21 mm (*p* ≤ 0.01) on the left side, and a condylar volume of 111.03 mm^3^ (*p* ≤ 0.01) on the right and 127.80 mm^3^ (*p* ≤ 0.001) on the left side. The Herbst group showed anterior remodelling on the postero-superior aspect of glenoid fossa. Herbst appliance treatment induced growth at the condylar head and anterior remodelling of glenoid fossa, thereby improving the maxilla-mandibular relationship in growing skeletal Class II patients.

## 1. Introduction

Class II malocclusion is a commonly encountered and treated malocclusion in orthodontic practice, with mandibular skeletal retrusion being the most common characteristic [1]. Class II disharmony does not tend to self-correct with growth and requires an intervention for correction of the underlying skeletal discrepancy. In growing patients, growth modification of skeletal structures, achieved by functional appliances, offers an intermediate treatment option in which the patient is intercepted when there is still growth to correct the skeletal discrepancy [2,3]; the ideal time for Class II growth modification is reported to be during pubertal growth spurt [4,5]. Functional appliances are basically of two types: removable and fixed functional appliances.

Removable functional therapy rely completely on patient compliance in wearing the appliance, for successful treatment. Fixed functional appliances such as Herbst, Forsus, Jasper Jumper, etc. have a distinct advantage as they eliminate patient compliance factors and deliver continuous forces. Treatment with the Herbst appliance can be successfully accomplished within a shorter duration of six to eight months, providing flexibility in the selection of treatment time during pubertal growth period [6,7,8,9,10].

Adaptive changes in condyle and glenoid fossa occur after Herbst therapy. However conventional cephalometric techniques used to evaluate these changes, provides only a two-dimensional representation of structures in three planes of space [11,12]. In addition to redirecting mandibular growth pattern, altering growth process of glenoid fossa also causes increased mandibular projection [12]. Condylar positional changes within the fossa have also been proposed but have not been significantly confirmed in either animal or human studies. Recent 3D studies on Herbst therapy have widened the scope of evaluating positional changes of condyle [11,13,14]. Further, several methodological flaws still exist, recommending additional 3D investigations for a thorough understanding of the effect of functional appliances on TMJ [15]. Translation of glenoid fossa has been shown to contribute to mandibular positional changes post Herbst treatment [11]. However, 2D imaging techniques used in human studies are greatly flawed when assessing for remodelling of glenoid fossa.

Cone beam computed tomography (CBCT), specifically developed for imaging maxillomandibular region, promises a true paradigm shift from two-dimensional to three-dimensional approach to data acquisition and image reconstruction [16]. It provides volumetric information for development of virtual 3D models which aids in visualising temporomandibular joints and diagnosing any asymmetry in complex craniofacial patterns. Hence, the present study was designed to compare the effects of Herbst appliance on the condyle and glenoid fossa complex of growing Class II division 1 patients with respect to growing Class II division 1 patients treated with fixed therapy and elastics.

## 2. Materials and Methods

This retrospective observational study was conducted at Department of Orthodontics and Dentofacial Orthopaedics, Faculty of Dental Sciences, from November 2015 to August 2017; after approval from Institutional Ethical committee (SGTU/FDS/24/1/717). Informed consent was obtained from all patients.

Considering the condylar height as main outcome, the study sample was calculated at power 80%, alpha level 0.5, and anticipation of large effect size (0.8), due to results of previous studies [17]. Based on this, power analysis showed a total sample size of 28 was required, with 14 subjects in each group. This number was increased to 15 in each group and thus a total of thirty subjects were included in the study. Patients in the age group 12–16 years, with skeletal Class II jaw relationship (ANB > 5), full cusp Class II molar and canine relationship, overjet 5–7 mm with minimal crowding in dental arches, normodivergent patients (22 < FMA < 30; 19° < PP-MP < 31°) reporting to Department OPD, were evaluated for Herbst appliance therapy. 15 patients (7 male; 8 female) (mean age: 13 years 2 months) who met the inclusion criteria were selected for this prospective study (Group 1), to be treated with Herbst appliance. A well- matched group of 15 Class II subjects (7 male; 8 female) (mean age: 14 years 5 months) treated with orthodontic camouflage with Class II elastics and fixed therapy without orthopedic force, were obtained from previous department database (Group 2). Descriptive statistics for Group 1 and Group 2 are summarized in Table 1 and Table 2, that showed that there was no statistically significant difference in pre-treatment craniofacial morphology and condyle-glenoid fossa variables used in the study, except for lower anterior facial height, which was less in Group 1. Patients with a history of treatment with other functional appliances, vertical growth pattern, end-on molar and canine relation, facial asymmetry, temporomandibular joint disorders, and craniofacial anomalies were excluded.

**Figure 1:**
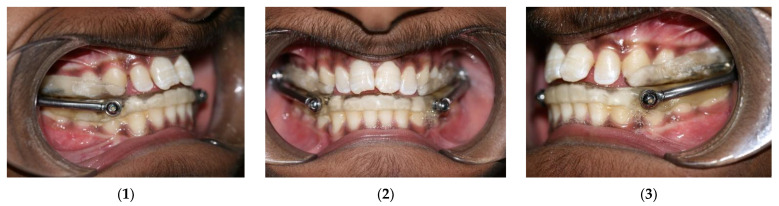
Herbst appliance: (**1**) right intraoral; (**2**) frontal intraoral; and (**3**) left intraoral.

Group 1 subjects were treated with acrylic splint Herbst appliance [18,19], cemented to dentition, keeping mandible forwardly postured to an edge-to-edge bite (Figure 1(1–3)). Patients were regularly evaluated for improvement in profile and correction of molar and canine relation. The average treatment time with Herbst appliance was 8–10 months, and treatment ended when Class I molar and canine relation was achieved. Group 2 subjects who had been previously treated with orthodontic camouflage using MBT prescription 0.022” slot (MBT^TM^ Versatile+; 3M, St Paul, MN, USA), and no orthopaedic force, had their pre- and post-treatment CBCT scans available in the department databank with an average time interval of 14–16 months at the end of their treatment.

**Figure 2:**
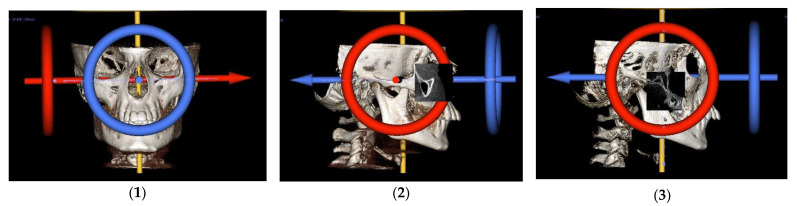
Bilaterally oriented 3D reconstructed CBCT image: (**1**) frontal view showing grid passing through right and left orbitale in transverse plane; (**2**) lateral view showing grid passing through right porion and right orbitale in transverse plan; and (**3**) lateral view showing grid passing through right and left Pterygomaxillary fissures in coronal plane.

Scans were carried out with I-CAT Cone Beam 3D Dental Imaging system (I-CAT Classic, Imaging Sciences International, Hatfield, PA, USA), operated at 90 kv and 14 mA with field of view (FOV) of 200 × 160 mm and voxel size of 0.3 mm. DICOM images were imported to In Vivo Dental 5.2.4 software (Anatomage, anatomy imaging software, San Jose, CA, USA). Scans from Group 1 and Group 2 were analysed by a single blinded examiner. The scans were standardised on volume rendered view using two reference planes, a transverse and a coronal plane (Figure 2(1–3)). For the transverse plane, the orientation grid was bilaterally placed through porion and orbitale on both right and left sides. For the coronal plane, the orientation grid was bilaterally placed tangent to posterior surface of right and left pterygomaxillary fissures (pterygoid vertical plane).

**Figure 3:**
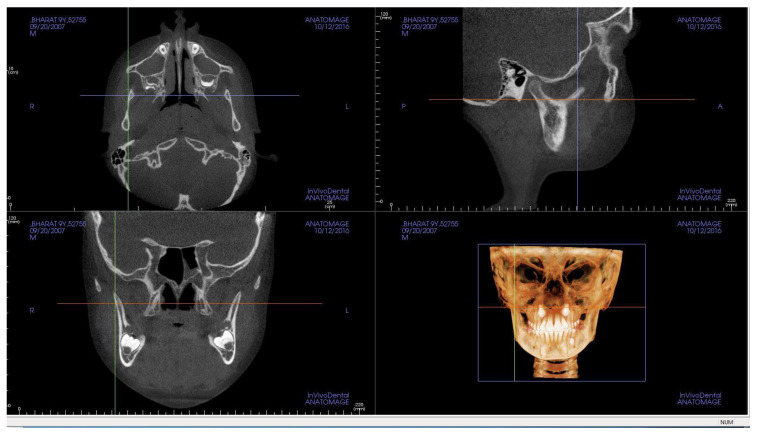
Standardization on MPR view for condyle-glenoid fossa measurements.

After orientation of the skull, standardization was carried out on MPR view to measure condyle-glenoid fossa variables. The Y-axis was set tangent through pterygoid vertical; the z-axis was placed along centre of sigmoid notch on axial section; and the x-axis (on sagittal section) was scrolled to be placed tangent to sigmoid notch (Figure 3). The procedure was followed for both right and left condyles and for all patients.

**Figure 4:**
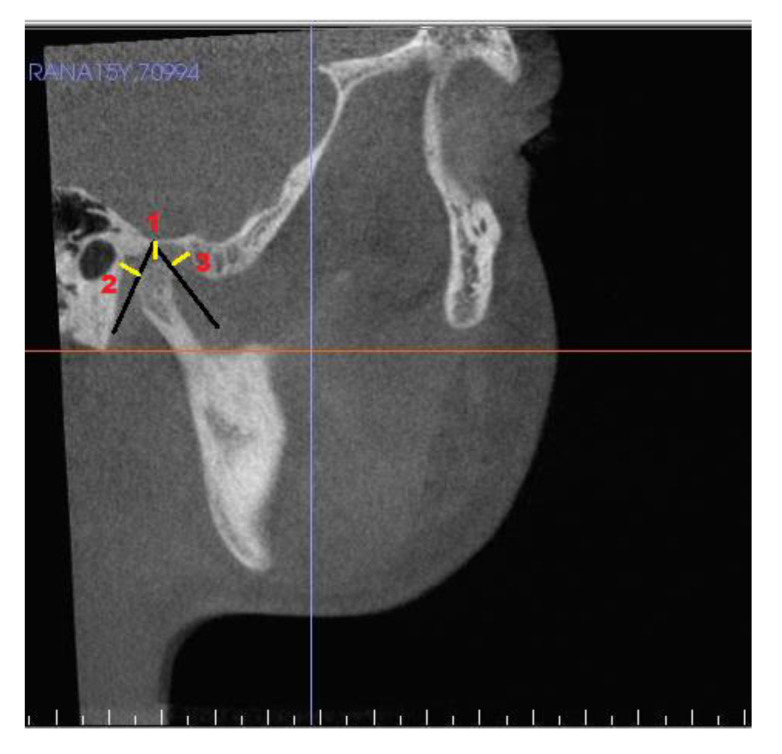
Joint space measurements: (**1**) superior joint space; (**2**) posterior joint space; and (**3**) anterior joint space.

**Figure 5:**
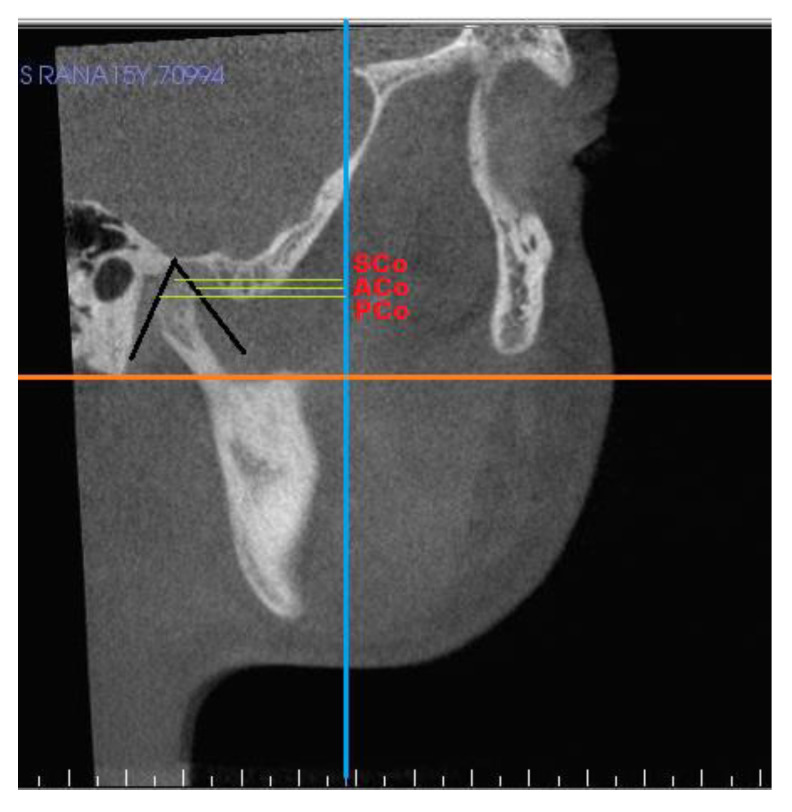
Condyle position measurements: SCo–T vertical; ACo–PT vertical; PCo–PT vertical.

**Figure 6:**
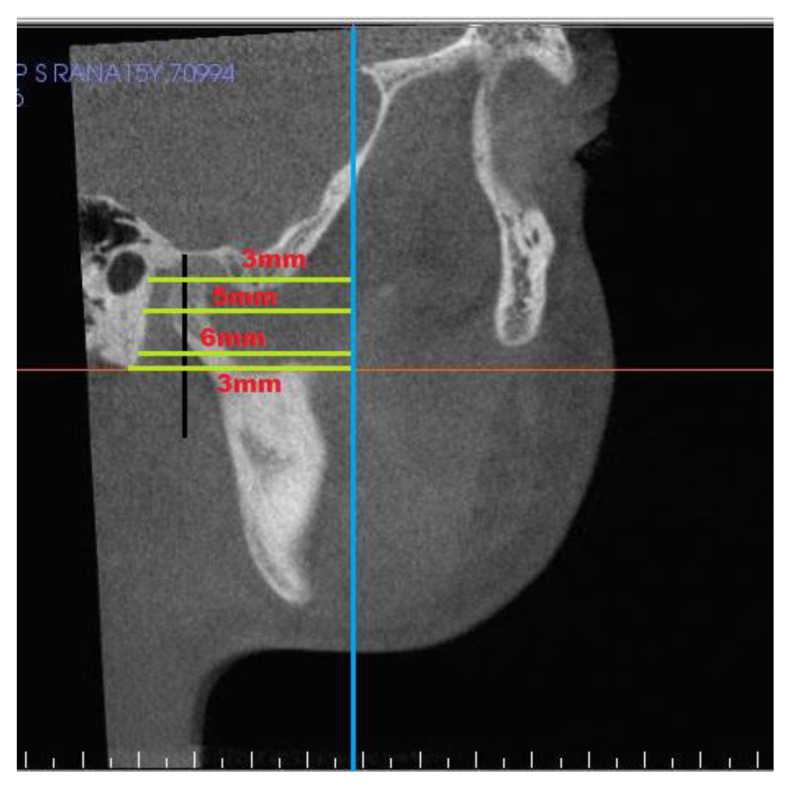
Posterior wall of glenoid fossa measurements: PF1—At distance 3 mm from superior point of fossa; PF2—At distance 5 mm from PF1; PF3—At distance 6 mm from PF2; PF4—At distance 3 mm from PF3.

**Figure 7:**
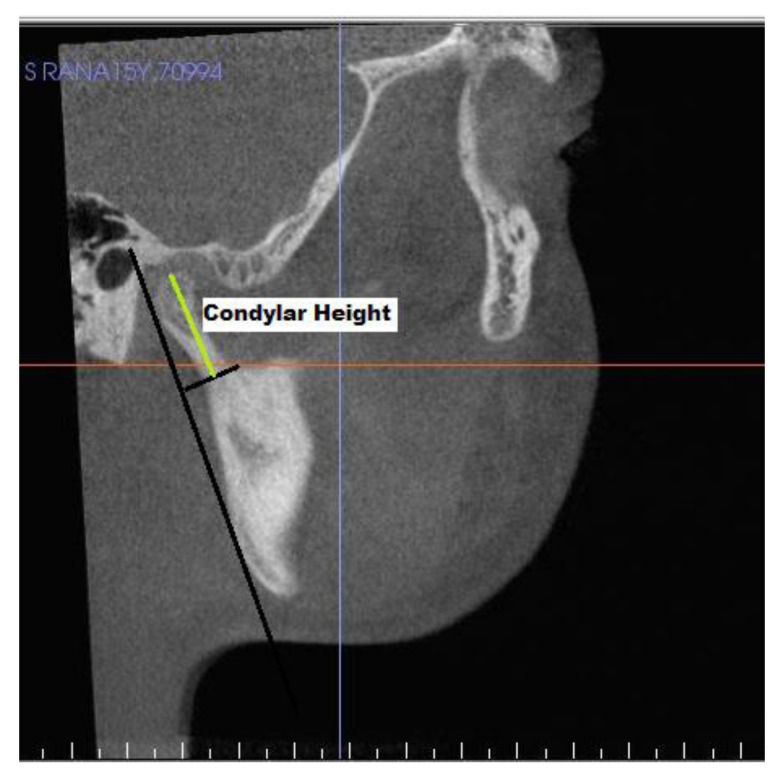
Condylar height measurement.

**Figure 8:**
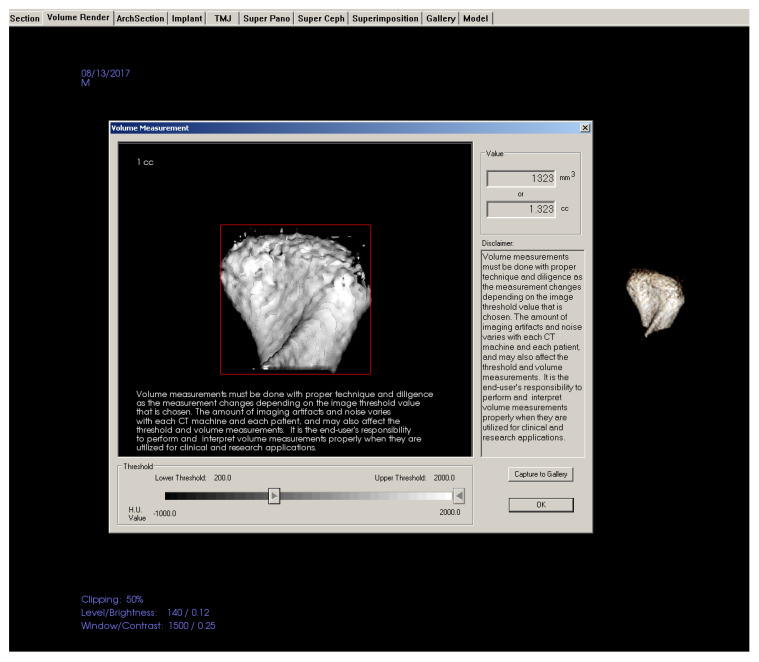
Condylar volume measurement.

Table 3 shows landmarks used for the evaluation of condyle-glenoid fossa changes. The position of the condyle was determined on sagittal view by calculating anterior, superior, and posterior joint space [20], (Figure 4) and linear distance of superior (SCo), anterior (ACo) and posterior (PCo) condylar points to pterygoid vertical plane (Figure 5). Figure 6, shows linear measurements from posterior wall of fossa divided into four sections of PF1 (3 mm), PF2 (5 mm), PF3 (6 mm), and PF4 (3 mm), relative to pterygoid vertical plane. Figure 7 shows the measurement of the height of the condyle. A sculpting tool was used to isolate the condylar head and its volume was calculated in mm3 using a volume measurement tool (Figure 8).

### Statistical Analysis

The software used for statistical analysis was SPSS (statistical package for social sciences) version 21.0 and Epi-info version 3.0. To check for intra-observer reliability, one CBCT scan was evaluated five times with a gap of three days and all of the parameters were retraced and remeasured by one investigator, then it was subjected to intra-class correlation coefficient (ICC) of reliability. To set the level of significance the Bonferroni correction for multiple testing was used (*p* < 0.05/11 = 0.004 for cephalometric analysis; *p* < 0.05/10 = 0.005 for changes in the condyle; *p* < 0.05/14 = 0.003 changes in the glenoid fossa) was used in this study. Pre-treatment skeletal values of both groups were subjected to an unpaired *t*-test to eliminate any bias between individual groups, and to check if all the patients in both the groups were well matched. All of the assessed variables were analyzed by means of Shapiro–Wilk test, to assess for normal distribution and then the inter-group comparison of mean difference between pre-treatment and post-treatment parameters in both groups was carried out using unpaired *t*-test.

## 3. Results

Descriptive statistics for Group 1 and Group 2 are summarized in Table 1 and Table 2. Statistically, the two groups were similar in craniofacial morphology. A high level of reproducibility of method of analysis was validated for each measurement with ICC, which was found to be between 0.809–0.935 showing a good agreement.

Table 4 shows an inter-group comparison between Group 1 and Group 2 for effective treatment changes in condyle. The mean difference in condylar height increase as seen in the Herbst group was by 1.35 mm (*p* ≤ 0.001) on the right side and 1.21 mm (*p* ≤ 0.01) on the left side. Considering changes obtained in Group 2, the volume of condyle in the Herbst group effectively increased by 111.03 mm^3^ on the right side and 127.80 mm^3^ on the left side. A negligible amount of increase in the condylar volume and height was seen in Group 2. Positional changes of the condyle were also determined by comparing linear distance of variables (i.e., posterior point (PCo), anterior point (ACo), and superior point (SCo)) on condyle with respect to the PT vertical, and no statistically significant change was seen post-treatment in both the groups (*p* ≥ 0.05).

Table 5 shows inter-group comparison between Group 1 and Group 2 for effective treatment changes in glenoid fossa. There were not statistically significant changes in the glenoid fossa.

## 4. Discussion

Skeletal Class II malocclusion develops early in deciduous dentition and does not tend to self-correct with age, implying that some sort of intervention is necessary to achieve correction [21]. Functional appliances, such as Herbst, have been purported to improve mandibular projection and translation of glenoid fossa/condyle complex, consequently improving the underlying skeletal discrepancies.

Longitudinal studies [21] comparing craniofacial growth changes in untreated Class II subjects with those having normal occlusion show a significant difference in mandibular growth between two groups and strongly suggest the need for untreated Class II malocclusions as controls in clinical studies on the mandibular effects of Class II treatment during the circumpubertal period [10,22]. In the present study, CBCT scans of subjects comprising Group 2, who met the inclusion criteria and were similar to the Herbst group in craniofacial characteristics, were obtained from department databank. This group was treated with orthodontic camouflage, however, without any orthopaedic force bringing about dentoalveolar changes with no skeletal enhancement, which justifies their use as control group for comparison of the changes observed in condyle-glenoid fossa complex in the two groups.

CBCT has not been frequently used in the evaluation of condylar response to functional orthopaedic therapy in patients with skeletal Class II malocclusion [23,24]. However, only recently, CBCT was used for the 3D assessment of mandibular and glenoid fossa changes [13,14,25,26]. It has been demonstrated that CBCT provides accurate and reliable linear measurement of the TMJ dimensions of dry human skulls. The measurement of joint spaces was also very similar to actual joint spaces [27,28]. Based on the ALARA principle “as low as reasonably achievable”, the potential benefits of diagnosis and treatment execution/ outcome must outweigh the potential risks of an increased radiation dose [29]. Considering the proven accuracy of CBCT, and variance in literature regarding skeletal changes produced by Class II orthopaedic therapy, this study was designed to quantify changes produced in condyle and glenoid fossa and to compare these findings with matched Class II subjects treated with orthodontic camouflage.

Changes in condylar dimensions and its position within glenoid fossa following Herbst therapy were measured and compared with changes obtained in Group 2. In comparison, the mean difference in condylar height increase seen in Group 1 was by 1.35 mm (*p* ≤ 0.001) on the right side and 1.21 mm (*p* ≤ 0.01) on the left side, which is suggestive of stimulation of condylar growth at superior border of condyle. In contrast, Group 2 showed a negligible amount of difference between the pre- and post-treatment values of condylar height (i.e., 0.16 mm on the right and 0.17 mm on the left side). An effective increase in condylar volume of 111.03 mm^3^ (*p* ≤ 0.01) on the right and 127.80 mm^3^ (*p* ≤ 0.001) on the left side was observed in Group 1. In contrast, Group 2 showed a negligible increase in condyle volume. This shows the increase in growth occurring at the condylar head due to its adaptive capacity in response to the 24 h forward positioning of mandible using Herbst therapy. It has been reported that Herbst appliance producing continuous forward mandibular positioning solicits cellular changes that enhance chondrogenesis and osteogenesis in condyles, resulting in true enhancement of condylar growth [30]. A short-term experiment demonstrated that hyper-propulsion brings about additional growth of condylar cartilage by stimulating pre-chondroblastic zone cells [31]. It was found that Herbst appliance treatment stimulated the condylar growth in the vertical direction [32]. The three-dimensional evaluation of skeletal mandibular changes following Herbst appliance have also shown greater 3D superior and posterior condylar growth than in their control group, resulting in significant mandibular forward displacement without pitch [14,26,33]. In the evaluation of changes in condylar volume, an average increase of 297 mm^3^ was reported in both the right and left condylar volumes in response to functional therapy with the twin block appliance [34].

In contrast to the above findings of increased condylar growth, a study revealed that in preadolescent Macaca fascicularis, condylar growth response was increased with Herbst treatment, but in adolescent animals there was no increase in condylar growth [35,36]. This study suggested that adaptive capability of adolescent monkeys and possibly adolescent humans might be chiefly limited to glenoid fossa with little potential for increased condylar length. Similarly, an MRI study observed structural changes in condyle with proliferation in postero-superior and reduction in anterior regions, albeit not validated on metric analysis [37]. Perhaps skeletal maturity could have greater and more direct influence on skeletal response to Herbst appliance than was previously understood.

Condylar positional changes were assessed by measuring distance by which condylar head moved with respect to reference plane (PT vertical). It was observed that the anterior point on mandibular condyle (ACo) showed a slightly forward displacement by 0.69 mm on the left and 0.75 mm on the right side, albeit not a statistically significant one. The difference in post-treatment and pre-treatment values of PCo (posterior point on condyle) and SCo (superior point on condyle) in Group 1 did not show displacement of these two points on both sides. Positional changes of condyle were also determined by evaluating changes in joint space. Quantitative measurements of joint space in pre- and post- treatment scans of Group 1 revealed an increase in superior joint space by 0.92 mm on the right and 1.27 mm on the left side with relatively no change observed in posterior joint space, suggesting that there was some vertical displacement of condyle due to the initial appliance placement. Condylar positional changes are in agreement with findings reported by Windmiller [38]. In the banded Herbst group, the appliance positions the condyle anteriorly against the eminence. In acrylic splint Herbst, however, the condylar position is much less forward initially and slightly displaced vertically. Voudoris et al., have shown condylar anterior condylar displacement following Herbst therapy is stabilized by addition of new bone in posterior aspect of fossa and increased fibrous tissue mass in the posterior aspect of disk [35,36]. The spatial orientation of condyle relatively remains unaffected within the fossa due to minor bone remodelling changes as well as the mechanical drift of condyle into its original position due to soft tissues traction [13,15].

The translation of glenoid fossa has been shown to contribute to mandibular positional changes after Herbst treatment in animal studies [30,35,36,39]. However, 2D imaging techniques used in human studies can have errors due to the difficulty involved in their assessment. We quantified remodeling changes occurring along the posterior wall of glenoid fossa as result of mandibular advancement therapy. The evaluation of posterior wall of glenoid fossa in Group 1 showed a slight reduction in linear distance of posterior wall of fossa relative to pterygoid vertical reference plane that was not statistically significant. This translation of glenoid fossa might contribute to anterior mandibular positional changes as well. However, in Group 2 there was an increase in linear distance between posterior wall of fossa and reference plane suggestive of posteriorly directed changes. This posterior repositioning of glenoid fossa is well documented [40,41]. During orthopaedic treatment, the fossa grows in a reverse direction, relocating antero-inferiorly to meet active condylar modification and to restore normal function. This relative restriction of normal fossa growth contributes toward Class II correction [21,42,43]. Remodelling on postero-superior surface of glenoid fossa seen in our study could be a result of pronounced adaptive capability of glenoid fossa relative to growing condyle [44]. Intensive remodelling changes have been reported on the caudal part of the post-glenoid spine and to lesser extent toward the fossa roof [45]. LeCornu stated that Herbst appliance alters the growth pattern of glenoid fossa, resulting in a more anteriorly positioned fossa and therefore more anterior position of mandible [11]. A sequence of cellular response and regional distribution of bone formation in the glenoid fossa has been quantified in response to mandibular forward positioning, providing evidence of a substantial increase in bone formation in treatment group when compared with untreated matched control rats [46]. Bone formation by mandibular advancement is triggered more in the posterior than anterior and middle regions of glenoid fossa, since primary attachment of posterior fibrous tissue to the articular disc occurs in this particular zone. In contrast, visual and metric analysis of parasagittal MRI slices showed no significant remodelling changes in glenoid fossa or articular eminence in response to functional mandibular advancer [37].

A major limitation of the study is small sample size that results in difficulty in comparing changes obtained using myofunctional therapy to that of growth changes. Also, availability of 3D data of untreated Class II control group could result in a well-designed study in future.

The study showed deposition at posterior wall of glenoid fossa and growth stimulation in Herbst treated subjects, with no significant change in position of condyle within fossa. Thus, there are simultaneous remodelling changes occurring in condyle and fossa resulting in improved mandibular anterior projection, thereby correcting the Class II jaw bases.

## 5. Conclusions

1. There was no significant positional change of condyle within glenoid fossa.
2. An increase in condylar volume showed enhanced growth at the condylar head in the Herbst treated group.

## Figures and Tables

**Table 1:** Shows pre-treatment statistical comparison for Group 1 and Group 2.

S. No.	Parameters	Group 1 N = 15		Group 2 N = 15		Mean Difference	Unpaired *t*-Test Value	*p*-Value
	Pre-Treatment	Mean	SD	Mean	SD			
1	ANB (deg)	7.65	1.93	6.20	2.26	1.45	1.723	0.098
2	A-PT vertical (mm)	52.13	3.28	49.57	2.99	2.56	1.974	0.061
3	B-PT vertical (mm)	42.30	2.93	42.40	5.19	−0.10	−0.063	0.950
4	Pog-PT vertical (mm)	44.30	3.23	43.57	6.02	0.73	0.392	0.699
5	WITS (mm)	5.40	1.71	3.29	1.95	2.11	2.869	0.009
6	Go-Gn (mm)	81.69	5.38	79.90	4.74	1.78	0.850	0.404
7	Co-Pog (mm)	107.52	5.97	106.82	2.44	0.70	0.350	0.730
8	Total anterior facial height (mm)	104.93	6.88	105.14	7.21	−0.21	−0.074	0.942
9	Lower anterior facial height (mm)	57.98	4.15	65.01	5.55	−7.03	−3.626	0.001 *
10	Total posterior facial height (mm)	67.12	3.82	61.03	8.18	6.09	2.518	0.019
11	FMA (deg)	23.90	3.76	25.28	8.37	−1.38	−0.563	0.579
12	PP-MP (deg)	20.82	2.62	24.12	6.10	−3.30	−1.8667	0.075

Not Significant—*p* > 0.004; Significant (*) *p* ≤ 0.004.

**Table 2:** Intergroup pretreatment comparison for condyle-glenoid fossa variables.

S. No.	Parameters	Side	Group 1		Group 2		Mean Difference	Unpaired *t*-Test Value	*p*-Value
	Pre-Treatment		Mean	SD	Mean	SD			
1	Condyle volume (mm^3^)	Right	864.67	167.59	841.30	213.80	23.37	0.306	0.762
		Left	955.00	234.90	867.70	207.31	87.30	0.952	0.351
2	Condyle height (mm)	Right	18.60	2.20	17.10	1.90	1.50	1.759	0.092
		Left	18.57	1.68	17.24	1.67	1.33	1.952	0.063
3	Condyle inclinationAngle (degrees)	Right	67.46	5.90	69.28	5.33	−1.82	−0.784	0.441
		Left	68.74	5.96	68.59	3.43	0.15	0.072	0.943
4	PCo-PT Vertical (mm)	Right	31.72	2.53	30.54	2.67	1.18	1.117	0.275
		Left	32.44	2.10	30.75	2.09	1.69	1.975	0.060
5	ACo-PT Vertical (mm)	Right	25.54	2.66	24.53	2.95	1.01	0.890	0.383
		Left	26.11	2.23	24.23	2.21	1.89	2.082	0.059
6	SCo-PT Vertical (mm)	Right	28.64	2.47	27.44	2.87	1.20	1.120	0.274
		Left	29.20	1.93	27.55	1.78	1.65	2.166	0.061
7	Superior joint space (mm)	Right	3.02	0.63	2.97	0.87	0.05	0.163	0.872
		Left	3.10	0.63	3.14	0.66	−0.04	−0.160	0.874
8	Posterior joint space (mm)	Right	2.69	0.53	2.87	0.65	−0.17	−0.736	0.469
		Left	2.49	0.64	2.90	0.66	−0.41	−1.539	0.137
9	Anterior joint space (mm)	Right	2.23	0.57	2.43	0.57	−0.20	−0.862	0.398
		Left	2.41	0.53	2.10	0.53	0.31	1.435	0.165
10	PF(a) [mm]	Right	33.95	2.54	32.01	2.86	1.94	1.782	0.088
		Left	34.33	2.37	32.09	2.73	2.24	2.178	0.060
11	PF(b) [mm]	Right	35.56	2.31	33.58	3.30	1.98	1.771	0.090
		Left	36.20	2.22	33.56	2.76	2.65	2.648	0.054
12	PF(c) [mm]	Right	36.63	2.71	34.89	4.15	1.74	1.276	0.215
		Left	37.68	2.78	35.39	3.27	2.30	1.885	0.072
13	PF(d) [mm]	Right	38.25	2.89	36.06	3.89	2.19	1.619	0.119
		Left	38.89	3.24	36.79	3.57	2.09	1.519	0.142

Not significant—*p* ≥ 0.004; Significant— *p* ≤ 0.004.

**Table 3:** Measurements used for evaluation of condyle-glenoid fossa changes.

S. No.	Measurments	Definition
	Condyle	
1	Superior joint space	Linear distance from superior point on condyle to highest point on glenoid fossa
2	Posterior joint space	Linear distance from posterior point on condyle to posterior surface of fossa
3	Anterior joint space	Linear distance from anterior point on condyle to a point on articular eminence
4	SCo–T vertical	Linear distance from superior point on condyle to pterygoid vertical
5	PCo–PT vertical	Linear distance from posterior point on condyle to pterygoid vertical
	Glenoid fossa	
6	ACo–PT vertical	Linear distance from anterior point on condyle to pterygoid vertical
7	PF 1 to PT vertical	At distance 3 mm from superior point of fossa
8	PF 2 to PT vertical	At distance 5 mm from PF1
9	PF 3 to PT vertical	At distance 6 mm from PF2
10	PF 4 to PT vertical	At distance 3 mm from PF3
11	Condylar height	Distance from SCo to constructed perpendicular line.
12	Condylar volume	Volumetric analysis of each mandibular condyle after isolating it.

**Table 4:** Inter-group comparison between Group 1 and Group 2 for effective treatment changes in condyle.

S. No.	Variable Measured	Side	Group 1		Group 2		Mean Difference	*t*-Test	*p*-Value
			T2-T1	SD	T2-T1	SD			
1.	Condyle volume (mm^3^)	right	147.13	96.13	36.10	42.46	111.03	3.418	0.002 *
		left	147.20	82.76	19.40	30.97	127.80	4.644	0.001 *
2.	Condyle height (mm)	right	1.51	1.14	0.16	0.24	1.35	3.674	0.001 *
		left	1.38	1.12	0.17	0.46	1.21	3.238	0.004 *
3.	PCo-PT Vertical (mm)	right	0.35	1.36	0.01	0.49	0.34	0.751	0.461
		left	−0.01	1.27	0.12	0.87	−0.13	−0.282	0.780
4.	ACo-PT Vertical (mm)	right	−0.75	1.48	0.22	0.55	−0.97	−1.966	0.062
		left	−0.69	1.26	−0.01	0.41	−0.68	−1.635	0.116
5.	SCo-PT Vertical (mm)	right	0.01	1.05	0.15	0.96	−0.13	−0.314	0.756
		left	−0.04	1.17	−0.16	0.92	0.12	0.265	0.793

PCo, posterior point on condyle; ACo, anterior point on condyle; SCo, superior point on condyle. Not Significant—*p* > 0.004; Significant (*) *p* ≤ 0.004.

**Table 5:** Inter-group comparison between Group 1 and Group 2 for effective treatment changes in glenoid fossa.

S. No.	Variable Measured	Side	Group 1		Group 2		Mean Difference	*t*-Test	*p*-Value
			T2-T1	SD	T2-T1	SD			
6.	Superior Joint SpaCE (mm)	right	0.92	1.38	0.09	0.49	0.83	1.811	0.083
		left	1.27	1.35	0.13	0.34	1.14	2.588	0.016
7.	Posterior Joint Space (mm)	right	−0.03	1.02	−0.04	0.38	0.01	0.021	0.984
		left	0.17	0.75	−0.09	0.30	0.26	1.046	0.306
8.	Anterior Joint Space (mm)	right	0.02	0.54	−0.04	0.22	0.05	0.300	0.767
		left	−0.23	0.49	0.10	0.13	−0.33	−2.061	0.041
9.	PF 1 [mm]	right	−0.63	1.25	0.27	0.49	−0.91	−2.179	0.040
		left	−0.78	1.35	0.26	0.37	−1.03	−2.351	0.028
10.	PF 2 [mm]	right	−0.29	0.84	0.00	0.27	−0.29	−1.049	0.305
		left	−0.75	0.89	0.15	0.49	−0.90	−2.891	0.008
11.	PF 3 [mm]	right	−0.25	1.22	−0.12	0.75	−0.13	−0.301	0.766
		left	−0.65	1.00	−0.07	0.58	−0.59	−1.663	0.110
12.	PF 4 [mm]	right	−0.67	1.69	−0.11	0.31	−0.56	−1.026	0.316
		left	−0.63	1.13	−0.04	0.42	−0.59	−1.572	0.130

PF, Posterior wall of fossa; PF 1—At distance 3 mm from superior point of fossa; PF 2—At distance 5 mm from PF1; PF 3—At distance 6 mm from PF2; PF 4—At distance 3 mm from PF3. Not Significant—*p* > 0.003; Significant—*p* ≤ 0.003.

## Data Availability

On request to corresponding author.

## References

[1] McNamara J.A. (1981). Components of Class II malocclusion in children 8–10 years of age. Angle Orthod..

[2] Bock N., Pancherz H. (2006). Herbst treatment of class II division 1 malocclusions in retrognathic and prognathic facial types. Angle Orthod..

[3] Hagg U., Pancherz H. (1988). Dentofacial orthopaedics in relation to chronological age, growth period and skeletal development: An analysis of 72 male patients with class II division 1 malocclusion treated with the herbst appliance. Eur. J. Orthod..

[4] Ruf S., Pancherz H. (1999). Dentoskeletal effects and facial profile changes in young adults treated with the herbst appliance. Angle Orthod..

[5] Ruf S., Pancherz H. (2006). Herbst/multibracket appliance treatment of class II division 1 malocclusions in early and late adulthood: A prospective cephalometric study of consecutively treated subjects. Eur. J. Orthod..

[6] Baccetti T., Franchi L., McNamara J.A. (2002). An improved version of the cervical vertebral maturation method for the assessment of mandibular growth. Angle Orthod..

[7] Kinzinger G., Kober C., Diedrich P. (2007). Topography and morphology of the mandibular condyle during fixed functional orthopedic treatment- a magnetic resonance imaging study. J. Orofac. Orthop..

[8] Pancherz H., Hägg U. (1985). Dentofacial orthopedics in relation to somatic maturation: An analysis of 70 consecutive cases treated with the Herbst appliance. Am. J. Orthod..

[9] Bock N.C., Jost J., Ruf S. (2021). Outcome quality of Class II division 1 Herbst-multibracket appliance treatment: Influence of pre-treatment Class II severity and skeletal maturity. Eur. J. Orthod..

[10] Croft R.S., Buschang P.H., English J.D., Meyer R. (1999). A cephalometric and tomographic evaluation of Herbst treatment in the mixed dentition. Am. J. Orthod. Dentofac. Orthop..

[11] LeCornu M., Cevidanes L.H., Zhu H., Wu C.D., Larson B., Nguyen T. (2013). Three-dimensional treatment outcomes in Class II patients treated with the herbst appliance: A pilot study. Am. J. Orthod. Dentofac. Orthop..

[12] Ruf S., Pancherz H. (1998). Temporomandibular joint growth adaptation in Herbst treatment: A prospective magnetic resonance imaging and cephalometric roentgenographic study. Eur. J. Orthod..

[13] Cheib Vilefort P.L., Farah L.O., Gontijo H.P., Moro A., Ruellas A.C.O., Cevidanes L.H.S., Nguyen T., Franchi L., McNamara J.A., Souki B.Q. (2019). Condyle-glenoid fossa relationship after Herbst appliance treatment during two stages of craniofacial skeletal maturation: A retrospective study. Orthod. Craniofac. Res..

[14] Wei R.Y., Atresh A., Ruellas A., Cevidanes L.H.S., Nguyen T., Larson B.E., Mangum J.E., Manton D.J., Schneider P.M. (2020). Three-dimensional condylar changes from Herbst appliance and multibracket treatment: A comparison with matched Class II elastics. Am. J. Orthod. Dentofac. Orthop..

[15] Al-Saleh M.A., Alsufyani N., Flores-Mir C., Nebbe B., Major P.W. (2015). Changes in temporomandibular joint morphology in class II patients treated with fixed mandibular repositioning and evaluated through 3D imaging: A systematic review. Orthod. Craniofac. Res..

[16] Scarfe W.C., Farman A.G. (2008). What is cone-beam CT and how does it work?. Dent. Clin. N. Am..

[17] Elfeky H.Y., Fayed M.S., Alhammadi M.S., Soliman S.A.Z., El Boghdadi D.M. (2018). Three-dimensional skeletal, dentoalveolar and temporomandibular joint changes produced by Twin Block functional appliance. J. Orofac. Orthop..

[18] Sidhu M.S. (1990). Fabrication and Management of splint design Herbst Appliance. J. Ind. Orthod. Soc..

[19] Sidhu M.S., Kharbanda O.P., Sidhu S.S. (1995). Cephalometric Analysis of changes produced by the Herbst Appliance in the treatment of Class II division 1 malocclusion. Br. J. Orthod..

[20] Leonardi R., Caltabiano M., Cavallini C., Sicurezza E., Barbato E., Spampinato C., Giordano D. (2012). Condyle fossa relationship associated with functional posterior crossbite, before and after rapid maxillary expansion. Angle Orthod..

[21] Stahl F., Baccetti T., Franchi L., McNamara J.A. (2008). Longitudinal growth changes in untreated subjects with Class II division 1 malocclusion. Am. J. Orthod. Dentofac. Orthop..

[22] Tulloch J.F.C., Phillips C., Koch G., Proffit W.R. (1997). The effect of early intervention on skeletal pattern in Class II malocclusion: A randomized clinical trial. Am. J. Orthod. Dentofac. Orthop..

[23] Atresh A., Cevidanes L.H.S., Yatabe M., Muniz L., Nguyen T., Larson B., Manton D.J., Schneider P.M. (2018). Three-dimensional treatment outcomes in Class II patients with different vertical facial patterns treated with the Herbst appliance. Am. J. Orthod. Dentofac. Orthop..

[24] Durão A.R., Pittayapat P., Rockenbach M.I.B., Olszewski R., Ng S., Ferreira A.P., Jacobs R. (2013). Validity of 2D lateral cephalometry in orthodontics: A systematic review. Prog. Orthod..

[25] De Clerck H., Nguyen T., de Paula L.K., Cevidanes L. (2012). Three-dimensional assessment of mandibular and glenoid fossa changes after bone-anchored Class III intermaxillary traction. Am. J. Orthod. Dentofac. Orthop..

[26] Fan Y., Schneider P., Matthews H., Roberts W.E., Xu T., Wei R., Claes P., Clement J., Kilpatrick N., Penington A. (2020). 3D assessment of mandibular skeletal effects produced by the Herbst appliance. BMC Oral Health.

[27] Hilgers M.L., Scarfe W.C., Scheetz J.P., Farman A.G. (2005). Accuracy of linear temporomandibular joint measurements with cone beam computed tomography and digital cephalometric radiography. Am. J. Orthod. Dentofac. Orthop..

[28] Zhang Z.L., Cheng J.G., Li G., Zhang J.Z., Zhang Z.Y., Ma X.C. (2012). Measurement accuracy of temporomandibular joint space in Promax 3-dimensional cone-beam computerized tomography images. Oral Surg. Oral Med. Oral Pathol. Oral Radiol..

[29] Garib D.G., Calil L.R., Leal C.R., Janson G. (2014). Is there a consensus for CBCT use in Orthodontics?. Dent. Press J. Orthod..

[30] McNamara J.A., Carlson D.S. (1979). Quantitative analysis of temporomandibular joint adaptations to protrusive function. Am. J. Orthod..

[31] Charlier J.P., Petrovic A., Stutzmann J.H. (1969). Effects of mandibular hyperpropulsion on the prechondroblastic zone of young rat condyle. Am. J. Orthod..

[32] Woodside D.G., Altuna G., Harvold E., Herbert M., Metaxas A. (1983). Primate experiments in malocclusion and bone induction. Am. J. Orthod..

[33] Souki B.Q., Vilefort P.L.C., Oliveira D.D., Andrade I., Ruellas A.C., Yatabe M.S., Nguyen T., Franchi L., McNamara J.A., Cevidanes L.H.S. (2017). Three-dimensional skeletal mandibular changes associated with Herbst appliance treatment. Orthod. Craniofac. Res..

[34] Yildirim E., Karacay S., Erkan M. (2014). Condylar response to functional therapy with Twin-Block as shown by cone-beam computed tomography. Angle Orthod..

[35] Voudouris J.C., Woodside D.G., Altuna G., Kuftinec M.M., Angelopoulos G., Bourque P.J. (2003). Condyle-fossa modifications and muscle interactions during Herbst treatment, Part 1: New technological methods. Am. J. Orthod. Dentofac. Orthop..

[36] Voudouris J.C., Woodside D.G., Altuna G., Angelopoulos G., Bourque P.J., Lacouture C.Y. (2003). Condyle-fossa modifications and muscle interactions during Herbst treatment, Part 2: Results and conclusions. Am. J. Orthod. Dentofac. Orthop..

[37] Kinzinger G., Hourfar J., Kober C., Lissan J.A. (2018). Mandibular fossa morphology during therapy with a fixed functional orthodontic appliance: A magnetic resonance imaging study. J. Orofac. Orthop..

[38] Windmiller E.C. (1993). The acrylic-splint Herbst appliance: A cephalometric evaluation. Am. J. Orthod. Dentofac. Orthop..

[39] Rabie A.B., Zhao Z., Shen G., Hägg E.U., Robinson W. (2001). Osteogenesis in the glenoid fossa in response to mandibular advancement. Am. J. Orthod. Dentofac. Orthop..

[40] Björk A. (1963). Variations in the growth pattern of the human mandible: Longitudinal radiographic study by the implant method. J. Dent. Res..

[41] Popovich F., Thompson G. (1977). Craniofacial templates for orthodontic case analysis. Am. J. Orthod..

[42] Baccetti T., Antonini A., Franchi L., Tonti M., Tollaro I. (1997). Glenoid fossa position in different facial types: A cephalometric study. J. Orthod..

[43] Buschang P.H., Santos-Pinto A. (1998). Condylar growth and glenoid fossa displacement during childhood and adolescence. Am. J. Orthod. Dentofac. Orthop..

[44] Voudouris J.C., Kuftinec M.M. (2000). Improved clinical use of Twin-block and Herbst as a result of radiating viscoelastic tissue forces on the condyle and fossa in treatment and long-term retention: Growth relativity. Am. J. Orthod. Dentofac. Orthop..

[45] Ruf S., Pancherz H. (1999). Temporomandibular joint remodeling in adolescents and young adults during Herbst treatment: A prospective longitudinal magnetic resonance imaging and cephalometric radiographic investigation. Am. J. Orthod. Dentofac. Orthop..

[46] Rabie A.B.M., She T.T., Hagg U. (2003). Functional appliance therapy accelerates and enhances condylar growth. Am. J. Orthod. Dentofac. Orthop..

